# Fetal malnutrition among neonates in African countries: a CAN score systematic review and meta-analysis

**DOI:** 10.1186/s12937-024-00989-3

**Published:** 2024-09-06

**Authors:** Ibsa Mussa, Adera Debella, Melat B. Maruta, Tamirat Getachew, Lemma Demissie Regassa, Mulugeta Gamachu, Alemayehu Deressa, Fethia Mohammed, Abdi Birhanu, Hamdi Fekredin Zakaria, Addis Eyeberu

**Affiliations:** 1 School of Public Health, College of Health and Medical Sciences, Haramaya University, Harar, Ethiopia; 2 School of Nursing and Midwifery, College of Health and Medical Sciences, Haramaya University, Harar, Ethiopia; 3 Department of Psychiatry, School of Nursing and Midwifery, College of Health and Medical Sciences, Haramaya University, Harar, Ethiopia; 4 Obstetrics and Gynecology, Menelik Hospital, Addis Abeba, Ethiopia; 5 School of Medicine, College of Health and Medical Sciences, Haramaya University, Harar, Ethiopia; 6 Department of Public Health, Rift Valley University, Harar, Ethiopia

**Keywords:** Fetal malnutrition, Newborns, Neonates, Africa

## Abstract

**Background:**

To reduce neonatal mortality, it is necessary to identify neonates with fetal malnutrition at birth using the clinical assessment score (CAN score). Furthermore, comprehensive summary data that shows burden of fetal malnutrition in Africa is scarce. As a result, this systematic review and meta-analysis aimed to assess fetal malnutrition among newborns in Africa.

**Method:**

The PRISMA guidelines were used for this study. Articles were obtained from databases and websites. The outcome of the study was fetal malnutrition, as determined using the CAN score. The meta-analysis of the primary and secondary outcomes was performed using Stata version 18 statistical software. The pooled prevalence with a 95% CI was estimated using the random effect method with the Der Simonian Liard model.

**Results:**

This meta-analysis and systematic review included 5356 newborns from 13 studies. The pooled prevalence of fetal malnutrition (FM) among newborns diagnosed using the CAN score in Africa was 19% [95% CI: 17, 22]. Based on subgroup analysis by publication year, the lowest prevalence of fetal malnutrition 17% (95% CI: 9–27) was observed in the studies published in the years 2020–2023. Maternal and fetal factors were significantly associated with fetal malnutrition.

**Conclusion:**

Nearly one-fifth of neonates delivered in Africa were found to have fetal malnutrition based on the clinical evaluation of nutritional status. It has also been established that maternal malnutrition, a lack of proper treatment during pregnancy, maternal malnutrition, and newborn morbidities were associated with fetal malnutrition. To prevent fetal malnutrition, integrated efforts should be made for early maternal infection screening. Furthermore, maternal nutritional therapy should be explored for malnourished pregnant women.

**Supplementary Information:**

The online version contains supplementary material available at 10.1186/s12937-024-00989-3.

## Introduction

Fetal malnutrition (FM) is defined as the loss or failure to acquire an appropriate amount of subcutaneous fat and muscles during pregnancy [1]. Fetal malnutrition is clinically distinguished by a variety of morphological defects, including thinner hair, reduced buccal and gluteal fat pads, a sagging, wrinkled neck, and accordion folds in the limbs. The nutritional status of newborns at delivery reflects the state of intrauterine fetal feeding. In undernourished babies, numerous systems are influenced, including tissue form and composition, metabolic processes, and enzyme activity [1, 2]. Newborns with FM are at greater risk of developing both early adverse outcomes (prematurity, hypoglycemia, hypothermia, convulsion, and low birth weight) [2] and late adverse outcomes (neurological handicaps, mental retardation, learning disorders, cardio-vascular diseases, and diabetic mellitus morbidities) [3, 4]. Whether or not they are small for their gestational age, neonates with FM are more likely to develop certain morbidities [5].

FM affects between 2% and 10% of all births worldwide, with developing countries having the highest incidence [6], On the other hand, the prevalence of fetal malnutrition among newborns in Africa, as determined using the CAN score, ranges from 13.5% in Nigeria [7] to 30% in Egypt [8].

The intrinsic capacity for the growth of the fetus and the mother’s ability to support that growth via the placenta are the fundamental regulators of fetal development. The mother offers substantially more developmental support than the fetus requires, and the fetus’s intrinsic ability dictates how much growth occurs during and after pregnancy [5, 9]. FM can be caused by poor maternal nutrition, the mother’s inability to digest and transmit sufficient nutrients, a decreased vascular and placental supply of nutrients to the fetus, and an increase in fetal demand due to rapid growth [10]. There is evidence that maternal risk factors for fetal malnutrition include advanced age, weight, height, parity, hypertension, poor obstetric history, antepartum hemorrhage, maternal illnesses, alcohol use, smoking, pre-pregnancy weight, and high altitude [5, 11, 12].

It is necessary to diagnose fetal malnutrition at delivery in order to identify neonates who are more vulnerable. The government might then use this information to design policies and undertake early interventions to reduce the morbidity and mortality rates associated with FM. Despite some national studies in various parts of the continent, there is no systematic review and meta-analysis study done on FM among newborns in Africa. The prevalence of FM varied significantly throughout these country-wide investigations. Hence, the current study was devoted to performing a systematic review and meta-analysis on fetal malnutrition among neonates in African countries, considering socioeconomic indices and maternal factors precipitation in Africa.

## Methods

### Protocol and eligibility criteria

This review was conducted to determine the overall prevalence of fetal malnutrition and associated factors among neonates in Africa based on the Preferred Reporting Items for Systematic Reviews and Meta-Analyses (PRISMA) guideline [13] [additional file 1].

Studies fulfilling the following eligibility criteria were considered in this study:

#### Populations

Newborns with their mothers in Africa.

#### Exposure

Factors associated with fetal malnutrition or determinants or risk factors of fetal malnutrition (such as sociodemographic characteristics, maternal nutritional status, socioeconomic factors, maternal health conditions, access to antenatal care). The comparators are newborns/mothers without identified risk factors for fetal malnutrition.

#### Context

Studies conducted in African countries.

#### Outcome

Fetal malnutrition.

#### Study design

Observational studies such as cross-sectional, cohort, and case-control studies that examined fetal malnutrition.

Furthermore, studies using clinical assessment of nutrition (CAN score) were included. All full-text papers were written in English and published from inception until June 18, 2023. Case series, reports, reviews, commentaries, and editorials were excluded from this review.

### Data sources and search strategy

The articles for this review were found using a combination of Boolean logic operators (AND, OR, NOT), Medical Subject Headings (MeSH), and keywords in electronic web-based searches on PubMed, EMBASE, Google Scholar, MEDLINE, SCOPUS, and Google Search. The search strategy for advanced PubMed includes fetal malnutrition “[All Fields] OR “fetal nutrition disorders “[MeSH Terms]. OR (“fetal” [All Fields] AND “nutrition” [All Fields] AND “disorders” [All Fields]) OR “fetal nutrition disorders” [All Fields] OR (“fetal” [All Fields] AND “malnutrition” [All Fields]) OR “fetal malnutrition” [All Fields]. Additionally, the above keywords were used to search articles using the names of each of the countries on the African continent in legitimate databases, websites, and institutional repositories.

### Study selection

The database search results were consolidated, and duplicate articles were manually removed using the reference management application (Endnote version X8 ^™^). The titles and abstracts of the papers were then carefully evaluated. Two writers (TG and AE) independently reviewed the full texts of the remaining publications to determine their eligibility based on predetermined inclusion and exclusion criteria. The objectives, methodology, population, and significant findings (prevalence/magnitude of FM and factors associated with/predictors/determinants of FM in Africa) of the full-text studies in English were then reviewed further. The two writers came to a logical agreement to handle any questions that developed during the extraction process, and the final agreement was finalized with the assistance of the author (AD).

### Data extraction

After locating relevant papers, the authors (TG and AE) extracted the data independently. Using a pre-defined Microsoft Excel 2016 sheet, data was retrieved from selected studies under the headings of author and year, region, setting, study design, sample size, study subject, data collection techniques, the principal result of interest, and determining factors. The accuracy of the data extraction was checked by comparing the results produced by the two writers. The quantitative information from the included articles was extracted, which included the overall sample size (N), frequency of occurrence (n), and effect size.

### Data item

The outcome variable of interest was fetal malnutrition. In this study, using the CAN score, a systematized observation of the characteristics of nine superficial physical parameters, including hair and buccal fat in the cheeks, chin, neck, arms, back inter or subscapular skin, buttocks, legs, chest, and abdominal wall skin, fetal malnutrition was diagnosed. The maximum and minimum total scores are 36 and 9, respectively. The severity of these symptoms was graded from 1 (worst, severe fetal malnutrition) to 4 (best, well-nourished). A score of < 25 was deemed clinical confirmation (CAN score) of fetal malnutrition in gestation [6].

### Methodological quality of studies

To assess the methodological quality of observational research (cross-sectional, case-control, and cohort studies), Newcastle-Ottawa Scale (NOS) for assessing the quality of non-randomized studies in meta-analyses tool was used [14]. The included studies’ methodological validity and the quality of their conclusions were scrutinized. The writers (TG, IM, AD, LD, and AE) independently assessed the quality of each study. The mean score of the two authors was utilized to make the final decision. To resolve the differences regarding the inclusion of the research, a consensus was established. Based on their performance against each tool indicator, the included studies were categorized as high, moderate, or low quality. Good quality is defined as 80% or more, moderate quality as 60–80%, and low quality as below 60%. The quality scores of the 13 studies ranged from 60 to 90%, with most studies (8 studies) scoring 90%. All 13 studies were considered of adequate quality for inclusion in the analyses. A critical appraisal is used to assess internal validity (systematic error) and external validity (generalizability) and to reduce the possibility of biases.

### Statistical analysis

Statistical analysis was conducted using the STATA version 18 statistical software. The meta-analysis data demonstrating the prevalence of fetal malnutrition in Africa were presented using forest plots. A meta-analysis of the fetal malnutrition was performed using a random effects model using the Der Simonian Liard method of analysis to reduce the heterogeneity of the included studies [15]. Subgroup analyses were also performed based on years of publication and countries. A meta-regression analysis was also performed to determine the causes of research heterogeneity. Furthermore, we also assess the source of heterogeneity using Galbraith plot. A meta-analysis of observational studies was performed based on the recommendations of the I^2^ statistic given by Higgins et al. [16] (an I^2^ of 75/100% and above, implying considerable heterogeneity). To identify potential publication bias, we utilized Egger’s regression test, trim fill analysis, and visual evaluation of a funnel plot [17]. Sensitivity analysis was performed to identify the effect of a single study on the overall estimate and to identify outliers.

## Results

### Search finding and risk of bias assessment

**Fig. 1:**
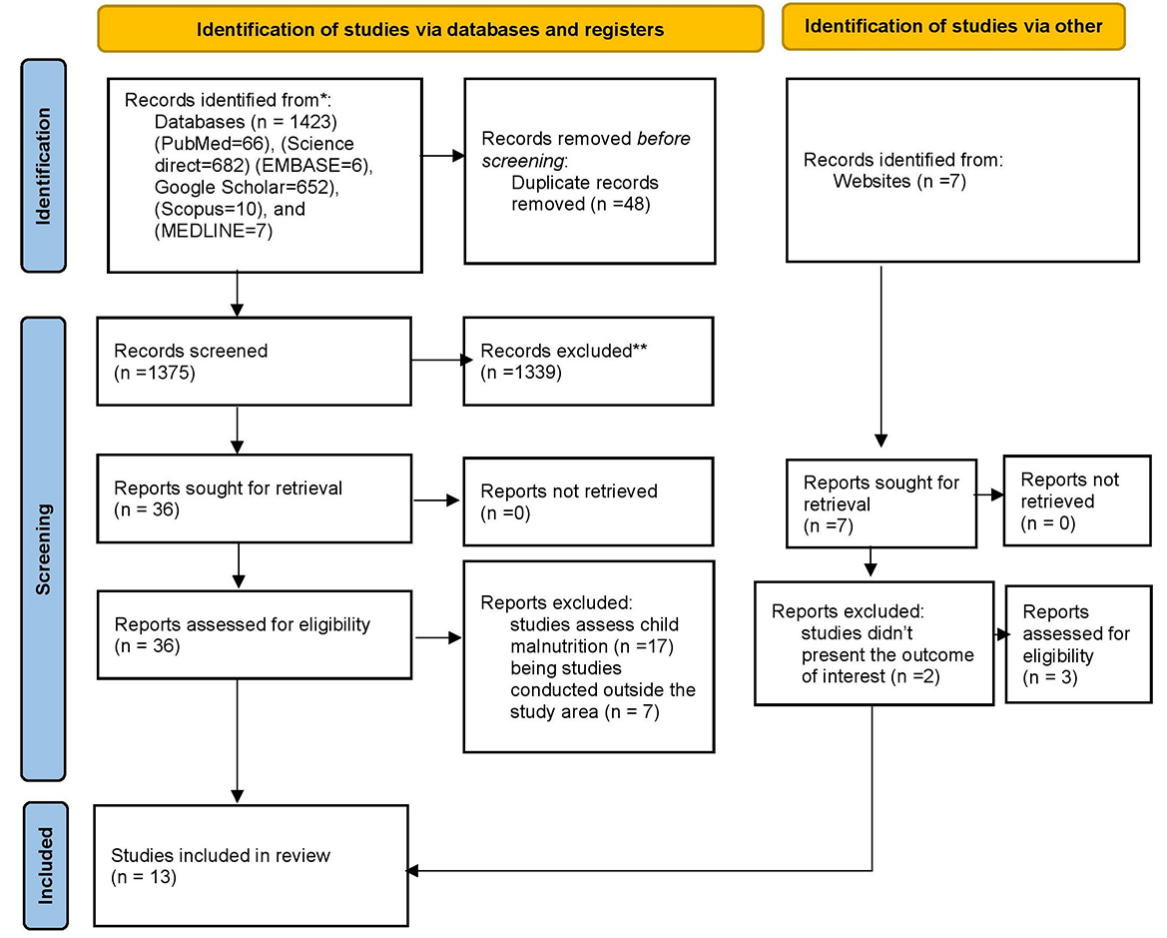
PRISMA 2020 flow diagram for systematic reviews and meta-analysis on fetal malnutrition among neonates in Africa, 2023

A total of 1430 articles were found in databases (1423) and websites (7). Using ENDNOTE and visual inspection, 48 publications found from databases were removed from all identified studies due to duplication. The remaining 1375 studies were then maintained and screened based on title and abstract. After being vetted based on titles and abstracts, 1339 were eliminated. From articles obtained from websites (7), four were eliminated after screened by title and abstract. Thirty-six (36) papers from databases and 3 articles from websites, a total of 39, were considered eligible. A total of 26 studies obtained from both databases and websites were removed because they measured child malnutrition and were done outside the region. Finally, the systematic review and meta-analysis comprised 13 observational studies from Ethiopia, Nigeria, and Egypt that met the inclusion criteria (Fig. 1). A thorough review of the included research across eight domains yielded high-quality scores.

### Characteristics of included studies

This systematic review and meta-analysis comprised 13 observational studies (cross-sectional and cohort studies) investigating fetal malnutrition among African neonates. The listed researches were published between 2007 [18] to 2023 [19]. Of all studies, three-fourths (77%) were from Western Africa [5, 18, 20–27], two studies from Eastern Africa [19, 28], and one study from Northern Africa [8]. All the included studies were observational (9 cross-sectional and 4 cohort studies). The sample size of the included studies ranged from 260 in a study done in Nigeria [21] to a maximum of 1592 in a study conducted in Ethiopia [28]. A total of 5356 newborns were included in this systematic review and meta-analysis. CAN score used in all studies to diagnose fetal malnutrition after delivery in hospital settings. Table 1 summarizes the main characteristics of the papers included in this systematic review and meta-analysis.

**Table 1:** Characteristics of included studies

No	Author and Years	Country	Setting	Design	Study subject	Diagnostic tool	Data collection method	Sample size	Outcome	Prevalence (%) along with 95% CI	Factors
1	Tesfa D, et al. 2021	Ethiopia	Hospital Based	Cross Sectional	Women and their newborns	Clinical assessment	Interview & Clinical assessment	1592	FM	21.7	Intimate partner violence (AOR: 1.97, 95% CI: 1.52–2.56), **placental weight less than 512 g (AOR: 2.76**,** 95% CI: 2.13–3.57)**, and **small for gestational age **(AOR: 1.96, 95% CI: 1.46–2.62)
2	Sume BW et al., 2023	Ethiopia	Hospital-Based	Cross-Sectional	Women and their newborns	Clinical assessment	Interview and clinical assessment	414	FM	12.32	ANC follow up (AOR: 3.47, 1.96–8.43), IFA provision (AOR:4.61, 1.23–10.63), dietary counseling (AOR: 6.01, 1.56–13.11), maternal MUAC < 22 cm (AOR: 2.49, 1.53–5.77), maternal BMI less than 18.5 kg/ m2 (AOR: 4.11,1.22–10.23), gestational age less than 37 weeks (AOR: 3.66, 1.89–8.49), gestational age > 42 weeks (AOR: 2.34,2.03–7.73), birth weight greater than 2.5 kg (AOR: 3.32, (1.52–7.44) and **placental weight less than** 519 g (AOR: 2.22,1.89–5.16)
3	Adebami OJ et al., 2007	Nigeria	Hospital-Based	Cohort	Newborn	Clinical assessment	Clinical assessment and chart review	473	FM and its problem	18.8	Meconium staining, sever asphyxia, RDS, hypoglycemia, Neonatal sepsis (p value less 0.05)
4	Ezenwa, B.N., 2012	Nigeria	Hospital-Based	Cross-Section	New born and mothers	Clinically	Clinical assessment, anthropometry. Review chart	422	Fm in term and preterm	13.5& 34.3	Low MAC and SGA
5	Ezenwa B, 2016	Nigeria	Hospital-Based	Cross-Section	New born and mothers	Clinically	Clinical assessment	282	Comparation of CAN score & Anthropometry of FM	14.5	
6	Adebami OJ et al., 2007	Nigeria	Hospital-Based	Cohort	New born	Clinically	Clinical assessment and review	304	Malaria and FM	21.7%	Malaria infection
7	Adebami OJ et al., 2007	Nigeria	Hospital-Based	Cohort	Newborn	Clinical assessment	Clinical assessment and chart review	473	Maternal factors of FM	18.8	lack of antenatal care, young mother (< 18 years), primiparity, maternal undernutrition (BMI < 18.5 kg/m(2) and MAC < 23.5 cm), low socioeconomic status, pregnancy-induced hypertension, antepartum hemorrhage, and maternal infections (p value less 0.05)
8	Tongo OO et al., 2013	Nigeria	Hospital	Cross-Sectional	Newborn	Clinically	Clinical assessment, review, and interview	322	FM using CAN SCORE AND Skin fold	20.2	
9	Rushid, 2018	Egypt	Hospital-Based	Cross-sectional	Newborn	Clinically	Clinical assessment	301	FM	29.9	
10	Josiah AE et al., 2018	Nigeria	Hospital-Based	Cohort	Newborn and their mother	Clinically	Clinical assessment and interview	300	FM	16.7	
11	Bolaji OB 2015	Nigeria	Hospital based	Cross sectional	Newborn and their mother	Clinically	Clinical assessment and interview	386	FM	23.3	hypozincemia
12	SafiyaYK, 2015	Nigeria	Hospital based	Cross sectional	Newborn and mothers	Clinically	Clinical assessment and record review	260	FM	24.6	
13	Opara p, 2019	Nigeria	Hospital based	Cross sectional	Newborn	Clinically	Clinical assessment,	300	Morbidity and weight gain in FM	17	

### Meta-analysis of prevalence of fetal malnutrition diagnosed using CAN score

**Fig. 2:**
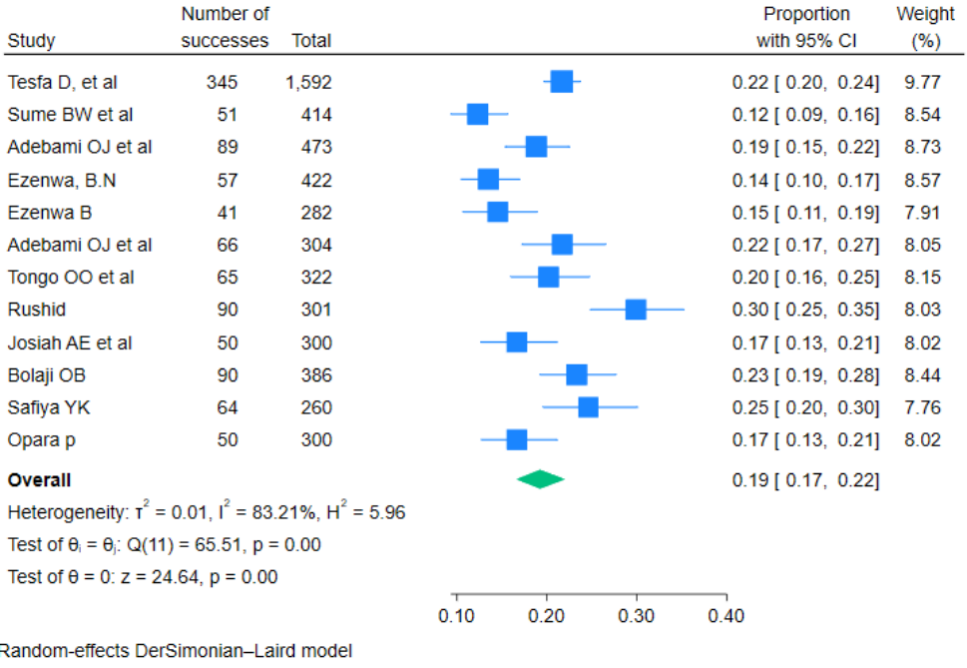
Pooled prevalence of fetal malnutrition among newborns diagnosed by CAN score in Africa, 2023

All of the thirteen studies report the prevalence of fetal malnutrition (FM) among newborns after delivery. The prevalence of FM ranges from 12% in a study done in Ethiopia by Sume BM et al. to 30% in Egypt by Rushid, 2018. The pooled prevalence of FM among newborns diagnosed by CAN score in Africa was 19% [95% CI: 17, 22] and heterogeneity; tau^2^ < 0.01, I^2^ = 83.21%; overall effect z = 24.64 (*p* < 0.001) (Fig. 2).

### Subgroup analysis of fetal malnutrition

**Fig. 3:**
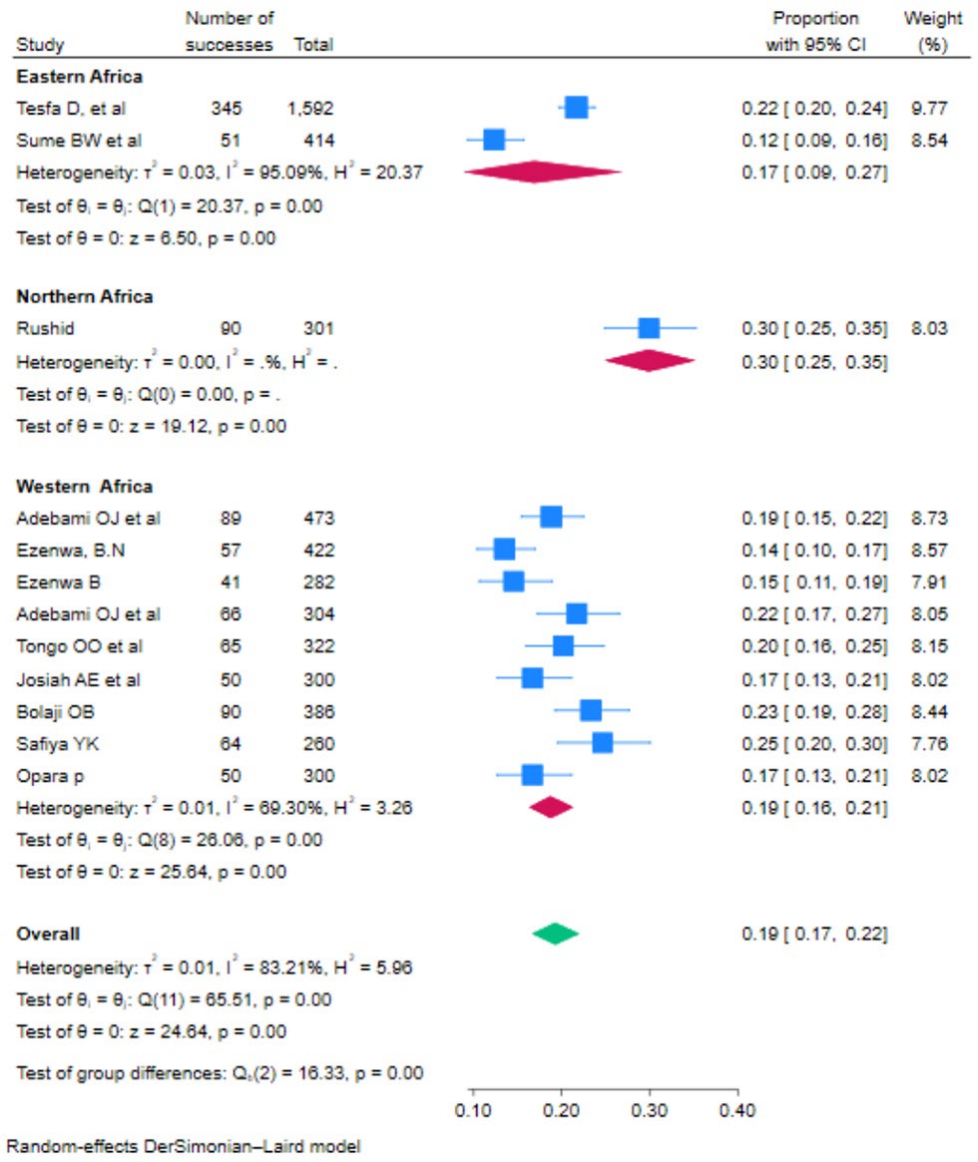
Subgroup analysis of the pooled prevalence of fetal malnutrition among newborns in Africa based on region, 2023

Based on subgroup analysis by the subcontinent where the studies were conducted, the lowest prevalence of FM among newborns 17% (95% CI: 9–27) and 19% (95% CI: 16–21) were observed among studies conducted in the eastern African countries (Fig. 3).

**Fig. 4:**
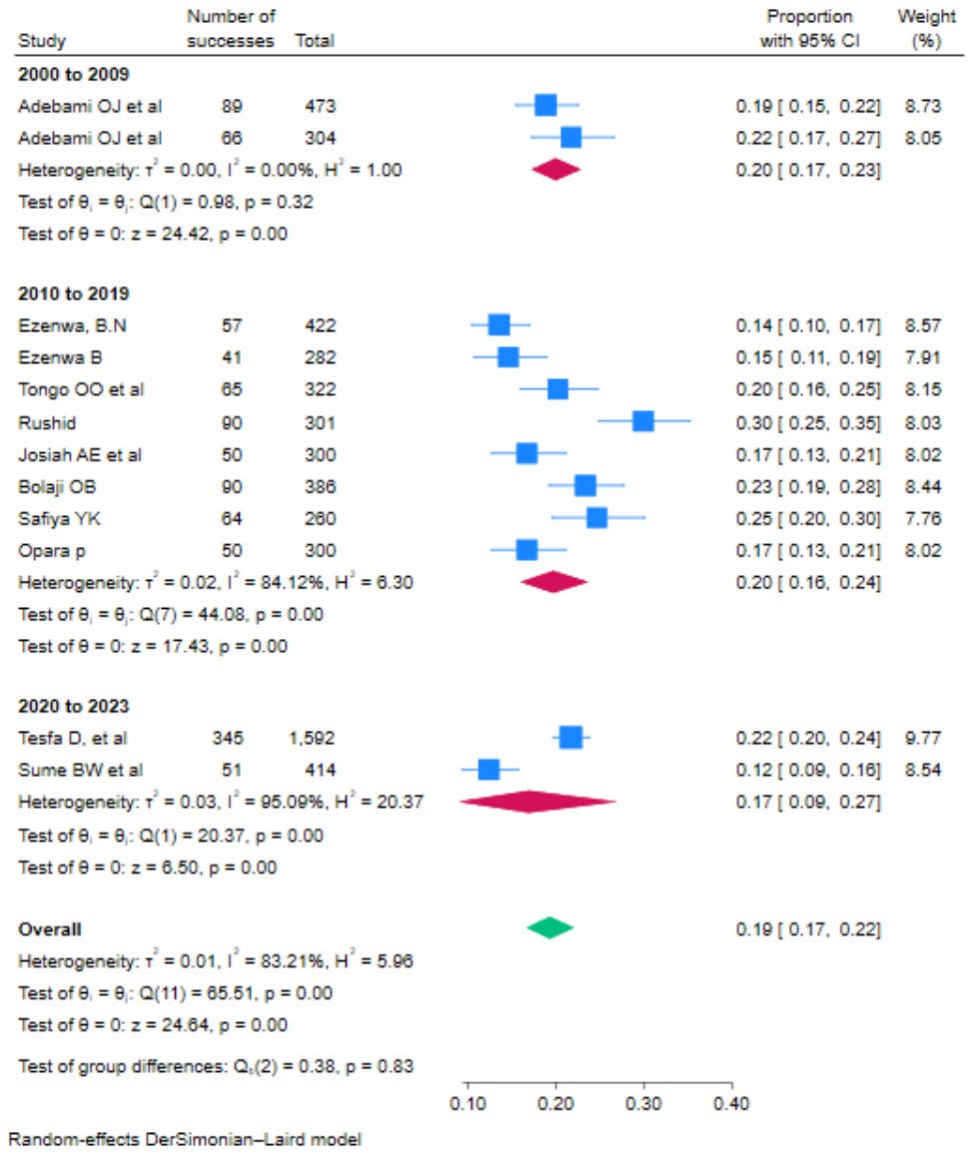
Subgroup analysis of the pooled prevalence of fetal malnutrition among newborns in Africa based on publication year, 2023

Based on subgroup analysis by publication year, the lowest prevalence of FM among newborns 17% (95% CI: 9–27) was observed in the studies published from 2020 to 2023 (Fig. 4**).**

### Publication bias

**Fig. 5:**
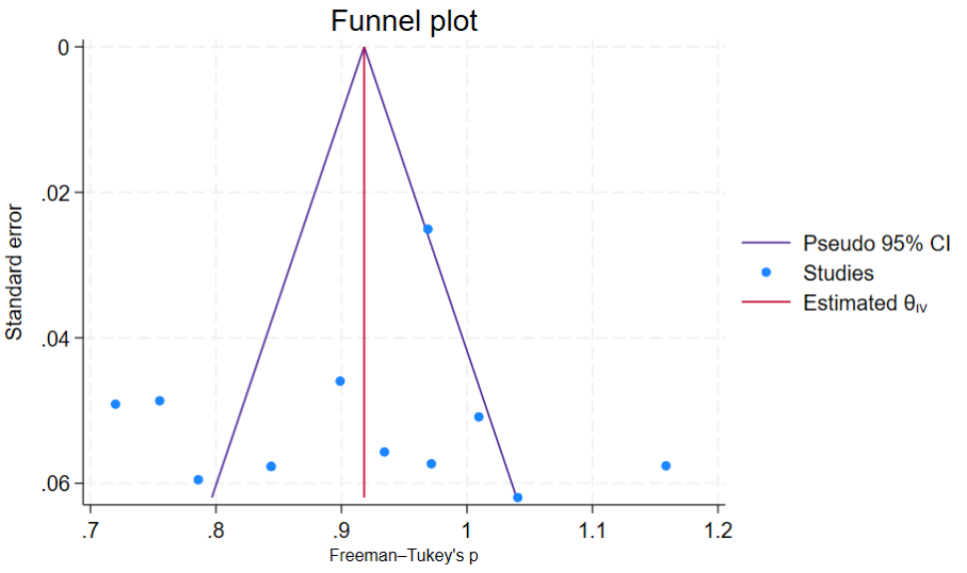
Funnel plot of fetal malnutrition among newborns diagnosed using CAN score in Africa, 2023

To observe publication bias, a visual inspection of the funnel plot was carried out. It shows that there is no one-sided view of the funnel plot but there are outliers on the right side of the plot (Fig. 5). Egger’s test showed that there was no small study effect on the estimate (*P* = 0.743). Furthermore, Trim fill analysis also showed no difference in observed or combination of observed and imputed effect size estimates in the random effect model utilizing Der Simonian Liard (additional file 2).

### Multivariate meta-regression

**Fig. 6:**
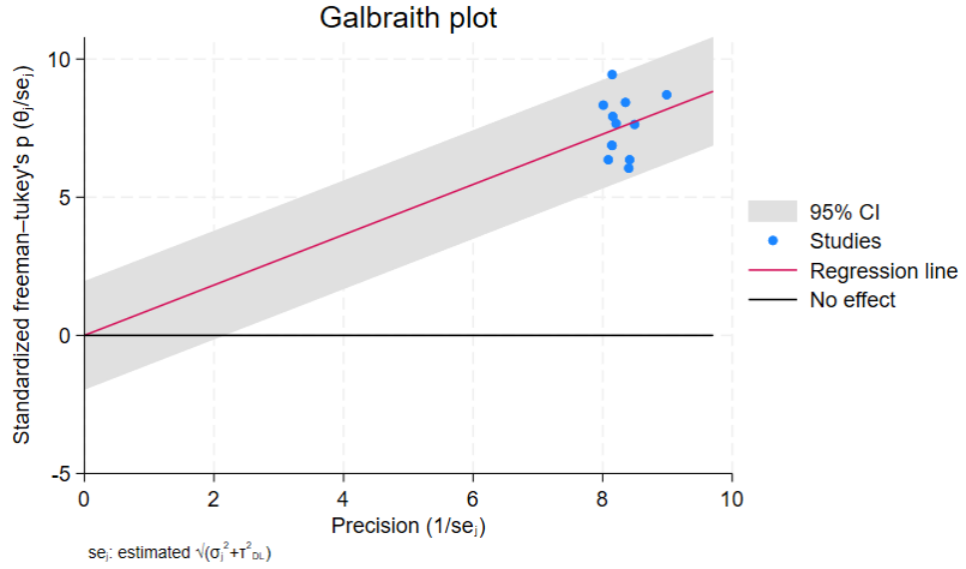
Galbraith plot of fetal malnutrition among newborns diagnosed using CAN score in Africa, 2023

Because there was statistically significant heterogeneity and the I-square test statistics were less than 0.001, a meta-regression analysis was performed. The aim of the analysis was to identify the source of heterogeneity so that the findings could be correctly interpreted. The meta-regression analysis showed that there were no significant variables identified that explain the source of heterogeneity. The study level covariates such as sample size and publication year of included studies were not statistically significant. As a result, the heterogeneity can be explained by aspects that these studies did not account for (Table 2).We also run the Galbraith plot (Fig. 6**)** to find the cause of heterogeneity. There were no studies found outside of the 95% confidence interval.

**Table 2:** Bivariate and multi variate meta-regression analysis to check heterogeneity, 2023

Variables	Coefficients	Standard error	*p*	95% CI
Publication year ^a^	-0.0012474	0 0.0031285	0.698	-0.0082181, 0.0057233
Sample size ^a^	0.0000123	0. 0000411	0.772	-0.0000794, 0.0001039
Publication year ^b^	-0.0017293	0. 0034609	0.629	-0.0095583, 0.0060997
Sample size ^b^	0.0000194	0.0000455	0.679	-0.0000834, 0.0001223

a = bivariate meta regression, b = multivariate meta regression

### Systematic review of factors

Of the total studies, seven articles report factors associated with fetal malnutrition among newborns in Africa [5, 18, 19, 24, 26–28]. Of the reported factors, fetal factors were significantly associated with fetal malnutrition. Placental weight below 519 g was significantly associated with fetal malnutrition, as reported by two studies in Ethiopia [19, 28]. A small gestational age was also found to be significantly associated with fetal malnutrition, as reported by studies done in Ethiopia [28] and Nigeria [24]. Other reported fetal factors, including prematurity, post maturity, low birthweight, respiratory distress syndrome, asphyxia, neonatal sepsis, and meconium aspiration syndrome were significantly associated with fetal malnutrition [18, 19].

Maternal factors were significantly associated with fetal malnutrition. Lack of antenatal care follow-up significantly associated with the nutritional status of the fetus, as reported by studies in Ethiopia and Nigeria [5, 19]. Maternal infections, including malaria, were found to be associated with fetal malnutrition [5, 27]. Other maternal factors such as intimate partner violence, folic acid supplementation, dietary counseling, maternal nutritional status, younger age, pregnancy complications (antepartum hemorrhage and hypertension), and low levels of zinc were also associated with fetal malnutrition among newborns in Africa [5, 26, 27].

#### Sensitivity analysis

**Fig. 7:**
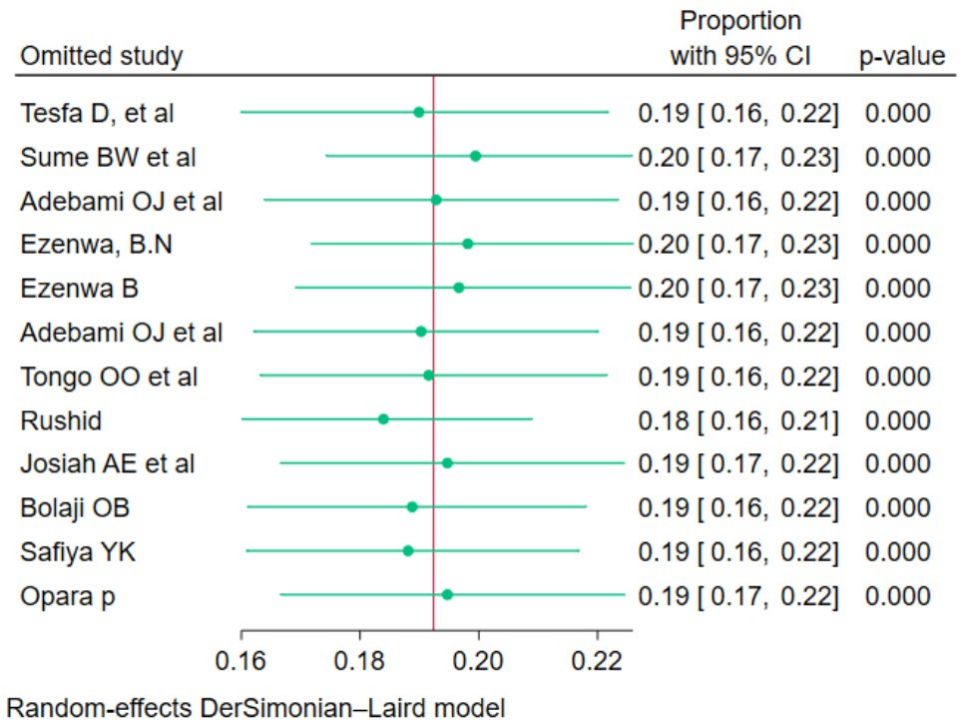
Leave one out meta-analysis of fetal malnutrition among newborns diagnosed using CAN score in Africa, 2023. REML = restricted maximum likelihood

Sensitivity analysis was assessed using a leave-one-out meta-analysis using the Der Simonian Liard method to determine the effect of a single study on the meta-analysis results and to identify outliers if they existed. As shown in the figure (Fig. 7), there was no significant difference observed when the outliers and inliers were removed.

## Discussion

This meta-analysis study demonstrated that the overall pooled prevalence of fetal malnutrition was 20% in African countries. The study findings showed a comparable magnitude of fetal malnutrition of 19.6% [29] and 16.3% [30] in different countries. Unfavorable fetal nutritional status can be caused by a variety of factors, including maternal body mass before and during pregnancy, placental weight below 519 g, and small gestational age [31]. Moreover, the government’s and other stakeholders’ nutrition intervention differences across countries can vary the level of fetal malnutrition burden from country to country [32, 33]. The present figure is lower than the one provided by India, which was 76.5% fetal malnutrition [34]. This finding demonstrated that the risky level differential between mothers and the variation in community-level poverty could cause these conclusions to diverge [35].

It has been demonstrated that fetal malnutrition in Africa is associated with several fetal factors. According to the review results of our study, prematurity, post maturity, low birth weight, respiratory distress syndrome, asphyxia, neonatal sepsis, meconium aspiration syndrome, and hypoxia have all been connected to FM. Reports on similar discoveries on other continents [36, 37]. This implies that malnourished newborns are more likely to encounter neurodevelopmental difficulties, illness, and mortality, all of which harm the current national and global goal of reducing neonatal mortality by 2030 [38]. Similar to this, post-maturity, small gestational age, and neonatal sepsis were all reported to be related to FM in this study. Thus, growth monitoring, follow-up, and prompt intervention for any newborn problems are all necessary for the reduction of neonatal morbidity and mortality [39]. Furthermore, infants with intrauterine growth retardation needed to have their development regularly noted as being less favorable than that of children with proper intrauterine growth before the introduction of intense prenatal and postnatal care, as well as early and high-calorie feeding [29].

Maternal variables such as a lack of antenatal care, malaria infection, intimate partner violence, folic acid supplementation, dietary counseling, maternal nutritional status, younger age, pregnancy complications (antepartum hemorrhage and hypertension), and low levels of zinc were also linked to FM in Africa. A previous study [29] also supported the finding that maternal factors contributed to FM. Because implementing and ensuring utilization of maternal health care services has the potential to be one of the most effective health interventions for preventing maternal and neonatal morbidity and mortality, care during pregnancy (increased coverage with care must be accompanied by improved quality of care to truly influence health outcomes) and maternal health conditions are critical indicators for fetal and neonatal health [40, 41]. Additionally, maternal health care provides opportunities for providing medical advice and assistance that can greatly improve the overall well-being of women and their unborn children [42]. So, this finding suggests that there is a need to improve the quality of maternal health care services and maternal health.

### Strengths and limitations of the study

The study’s strength is that the publications were found on various websites and institutional repositories. Another strength is that, to the investigators’ knowledge, it is the first SRM on fetal malnutrition diagnosed using the CAN score. The study’s limitations include that only the English language was considered for the search of articles.

## Conclusion

Using clinical assessment of nutritional status, almost one-fifth of newborns delivered in Africa were identified as having fetal malnutrition. It has been established that maternal malnutrition, a lack of proper treatment during pregnancy, maternal malnutrition, and newborn morbidities associated with fetal malnutrition. To prevent fetal malnutrition, integrated efforts should be made for early maternal infection screening. Furthermore, maternal nutritional therapy should be explored for malnourished pregnant women.

## Electronic supplementary material

Below is the link to the electronic supplementary material.

Supplementary Material 1

Supplementary Material 2

## Data Availability

In this study, all pertinent information is given. However, the corresponding author will provide more information upon reasonable request.
