# Correction: Fetal malnutrition among neonates in African countries: a CAN score systematic review and meta-analysis

**DOI:** 10.1186/s12937-024-01025-0

**Published:** 2024-10-08

**Authors:** Ibsa Mussa, Adera Debella, Melat B. Maruta, Tamirat Getachew, Lemma Demissie Regassa, Mulugeta Gamachu, Alemayehu Deressa, Fethia Mohammed, Abdi Birhanu, Hamdi Fekredin Zakaria, Addis Eyeberu

**Affiliations:** 1 School of Public Health, College of Health and Medical Sciences, Haramaya University, Harar, Ethiopia; 2 School of Nursing and Midwifery, College of Health and Medical Sciences, Haramaya University, Harar, Ethiopia; 3 Department of Psychiatry, School of Nursing and Midwifery, College of Health and Medical Sciences, Haramaya University, Harar, Ethiopia; 4 Obstetrics and Gynecology, Menelik Hospital, Addis Abeba, Ethiopia; 5 School of Medicine, College of Health and Medical Sciences, Haramaya University, Harar, Ethiopia; 6 Department of Public Health, Rift Valley University, Harar, Ethiopia

**Correction: Nutr J 23**,** 102 (2024).**

10.1186/s12937-024-00989-3

Following publication of the original article [1], it has been reported that one of the authors’ name and affiliation should be corrected.

The author.

Hamdi Fikradin.

School of Nursing and Midwifery, College of Health and Medical Sciences, Haramaya University, Harar, Ethiopia.

Should be.

Hamdi Fekredin Zakaria.

School of Public Health, College of Health and Medical Sciences, Haramaya University, Harar, Ethiopia.

Thank you.

The original article has been updated.
