# Supplementary figures and images for: Fetal malnutrition among neonates in African countries: a CAN score systematic review and meta-analysis
Source: Nutr J. 2024 Sep 6;23:102. doi: 10.1186/s12937-024-00989-3 (PMC11380204; doi:10.1186/s12937-024-00989-3)

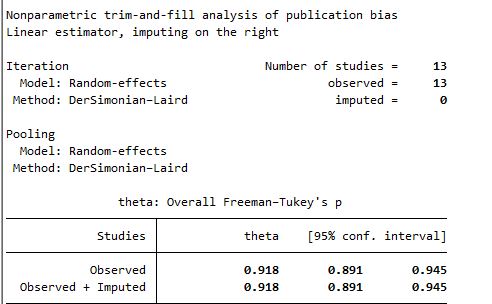

Supplement: Supplementary file 1 — Supplementary Material 1 [file 12937_2024_989_MOESM1_ESM.jpg]

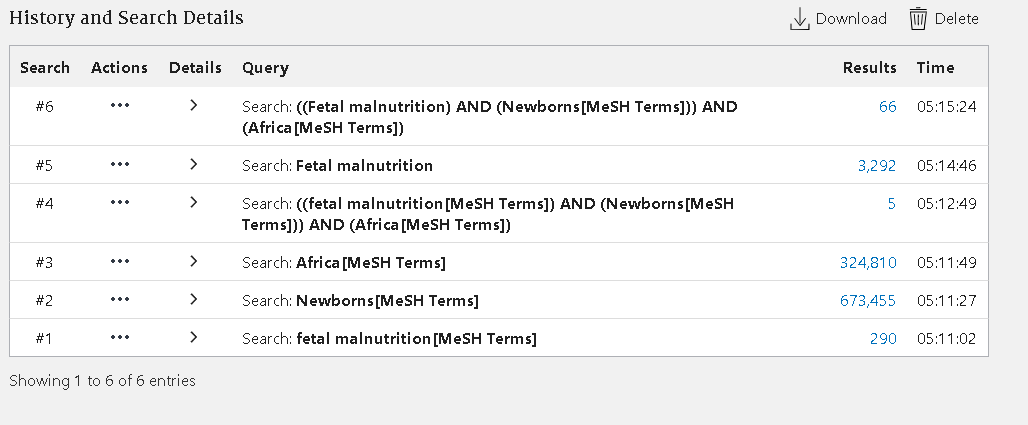

Supplement: Supplementary file 2 — Supplementary Material 2 [file 12937_2024_989_MOESM2_ESM.png]
